# Supplementary material for: Androgen Receptor Pathway Activity Assay for Sepsis Diagnosis and Prediction of Favorable Prognosis
Source: Front Med (Lausanne). 2021 Nov 23;8:767145. doi: 10.3389/fmed.2021.767145 (PMC8650119; doi:10.3389/fmed.2021.767145)
Supplement: Supplementary file 1 [file Data_Sheet_1.pdf]

Androgen receptor pathway activity assay for sepsis diagnosis and prediction of favorable prognosis

Wilbert Bouwman[1], Wim Verhaegh[1], Anja van de Stolpe[2]

[1]Philips Research, Eindhoven, Netherlands, [2]Philips Molecular Pathway Dx, Eindhoven, Netherlands

Supplementary information (I)

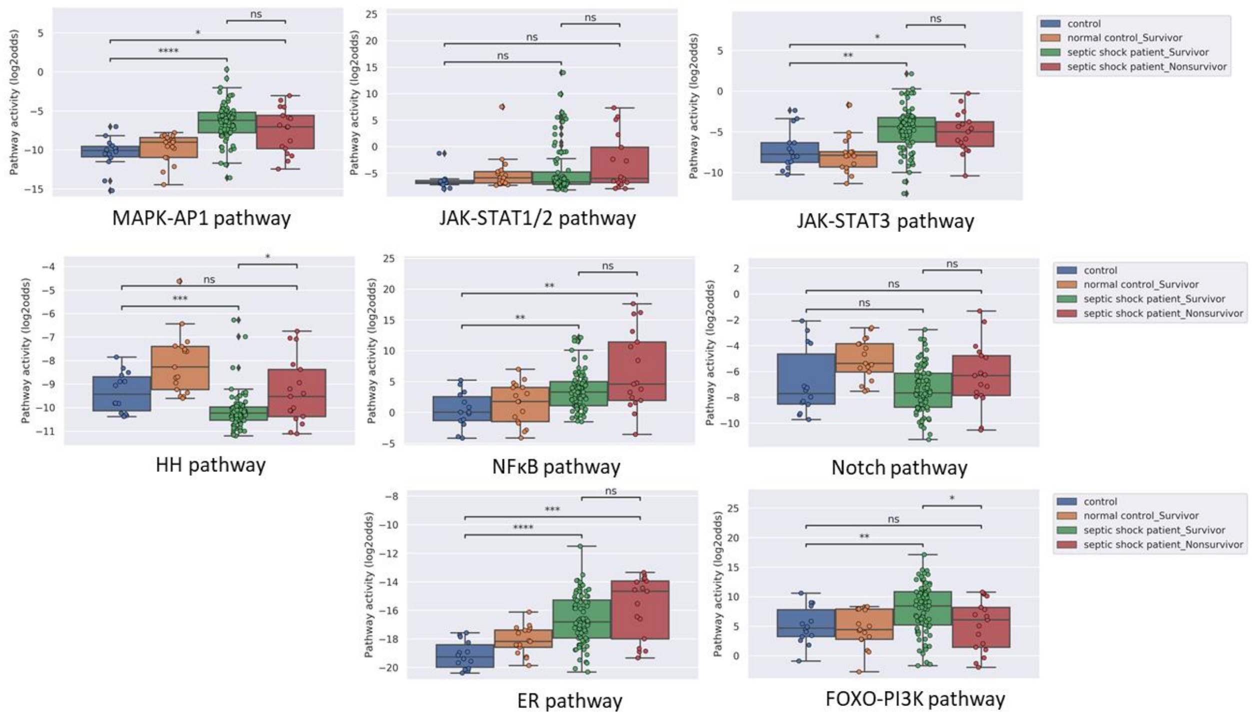

Figure S1: PAS for pathways NFκB, Notch, ER, FOXO, HH, MAPK-AP1, JAK-STAT3 and JAK-STAT1/2 of dataset GSE26440 (1). Pathway activity score on Y-axis in log2 odds. Two sided Mann–Whitney–Wilcoxon statistical tests were performed; p-values are indicated in the figures as *p <0.05, **p < 0.01, ***p < 0.001, ****p < 0.0001 or ns.

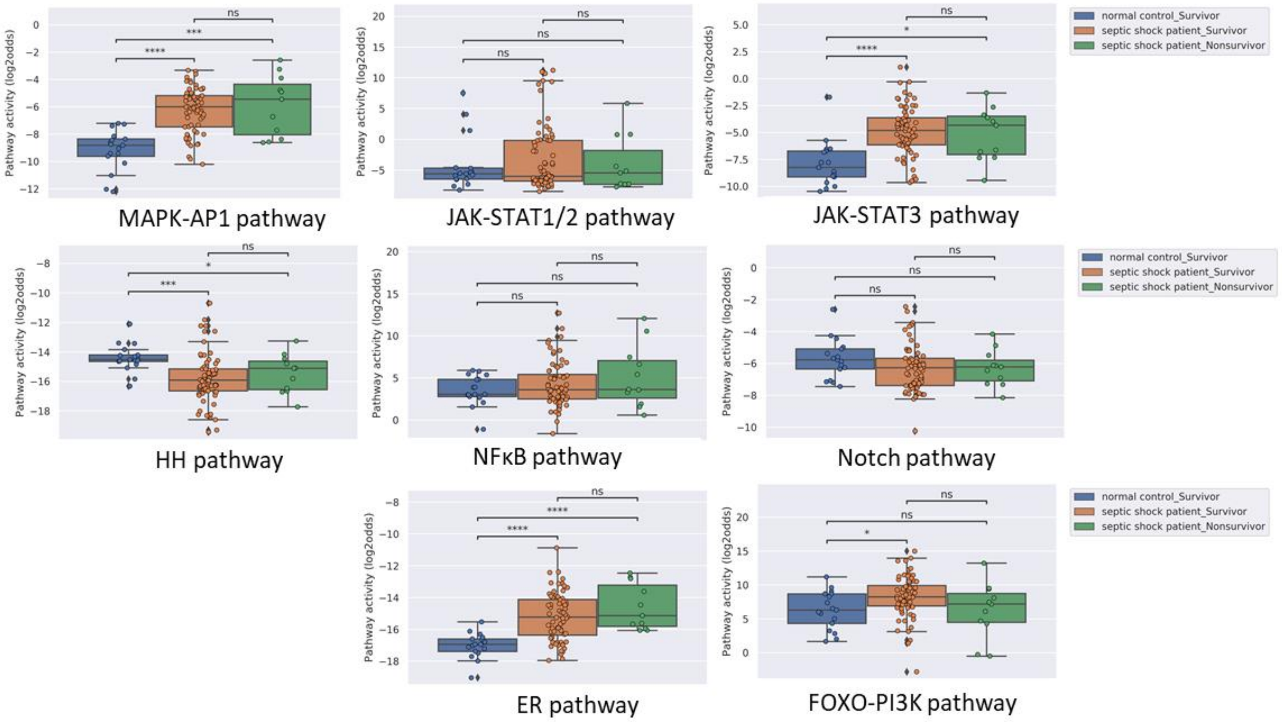

Figure S2: PAS for pathways NFκB, NOTCH, ER, FOXO-PI3K, HH, MAPK-AP1, JAK-STAT3 and JAK-STAT1/2 of dataset GSE26378 (1). *Two sided Mann–Whitney–Wilcoxon statistical tests were performed; p-values are indicated in the figures as *p <0.05, **p < 0.01, ***p < 0.001, ****p < 0.0001 or ns.*

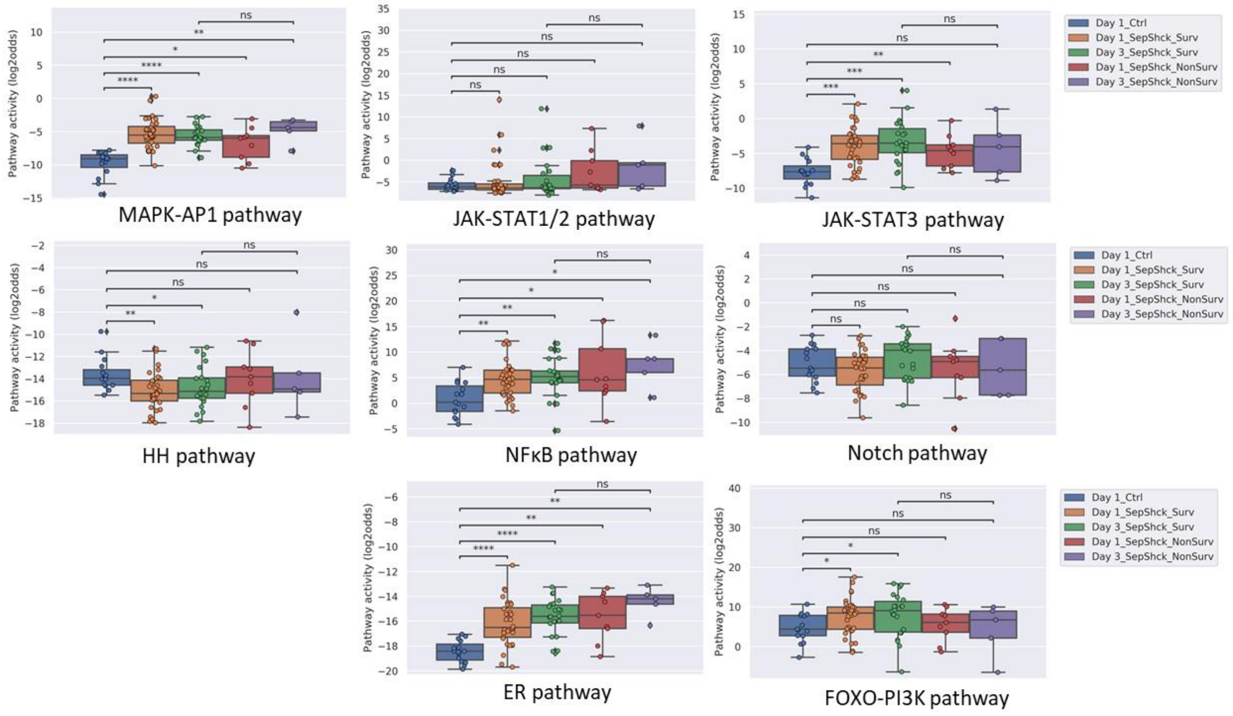

Figure S3: PAS for pathways NFκKB, Notch, ER, FOXO-PI3K, HH, MAPK-AP1, JAK-STAT3 and JAK-STAT1/2 of dataset GSE4607 (2). *Two sided Mann–Whitney–Wilcoxon statistical tests were performed; p-values are indicated in the figures as *p <0.05, **p < 0.01, ***p < 0.001, ****p < 0.0001 or ns.*

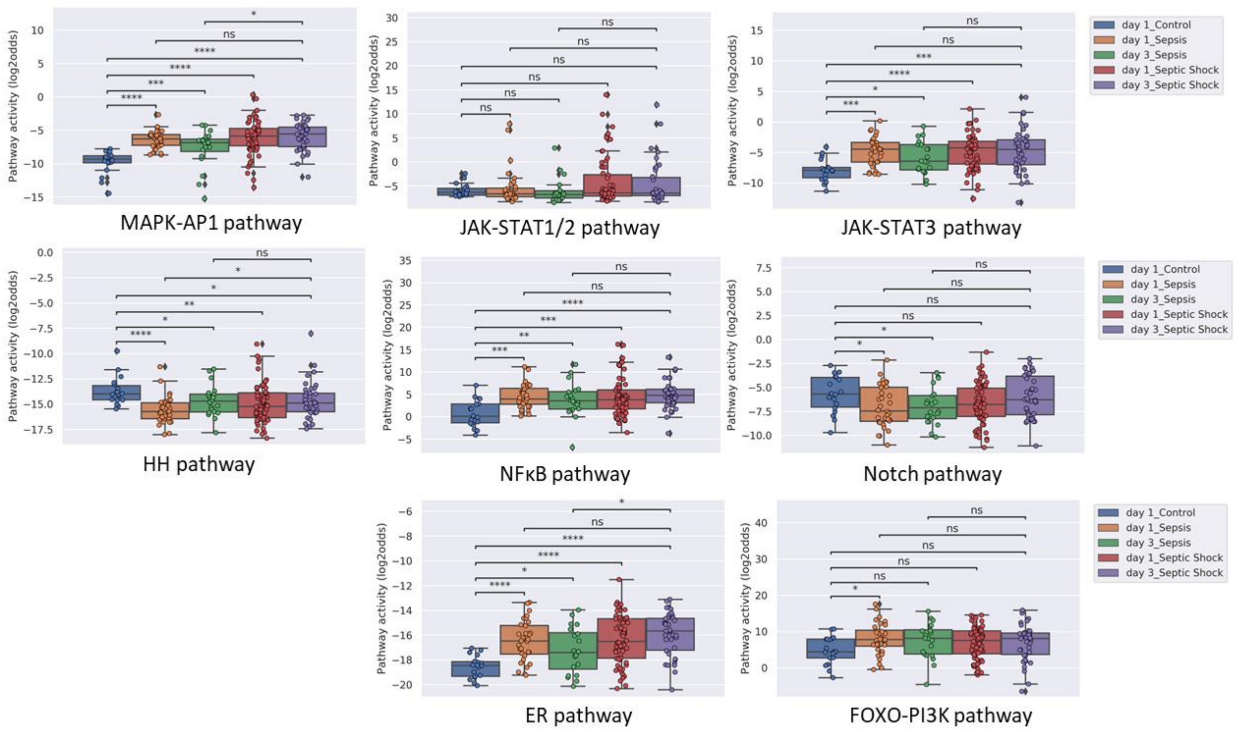

Figure S4: PAS for pathways NFκB, Notch, ER, FOXO-PI3K, HH, MAPK-AP1, JAK-STAT3 and JAK-STAT1/2 of dataset GSE13904 (3). *Two sided Mann–Whitney–Wilcoxon statistical tests were performed; p-values are indicated in the figures as *p <0.05, **p < 0.01, ***p < 0.001, ****p < 0.0001 or ns.*

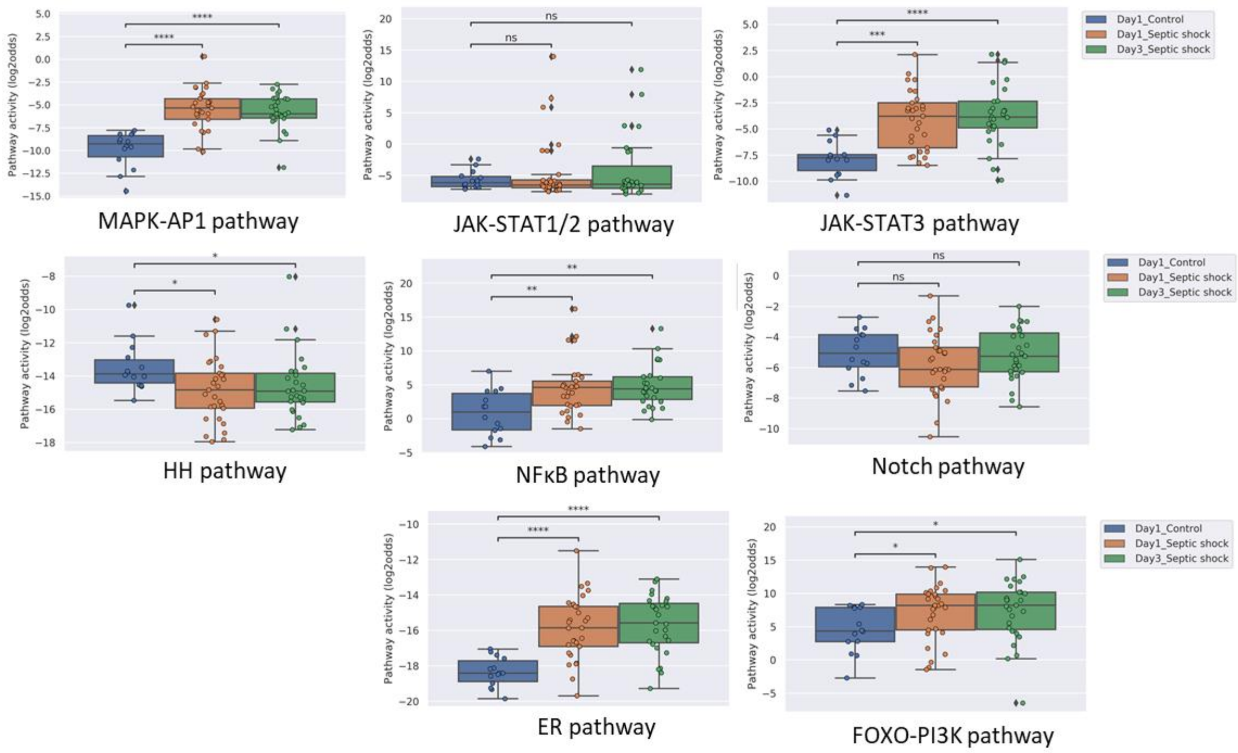

Figure S5: PAS for pathways NFκB, Notch, ER, FOXO-PI3K, HH, MAPK-AP1, JAK-STAT3 and JAK-STAT1/2 of dataset GSE8121 (4). *Two sided Mann–Whitney–Wilcoxon statistical tests were performed; p-values are indicated in the figures as *p <0.05, **p < 0.01, ***p < 0.001, ****p < 0.0001 or ns.*

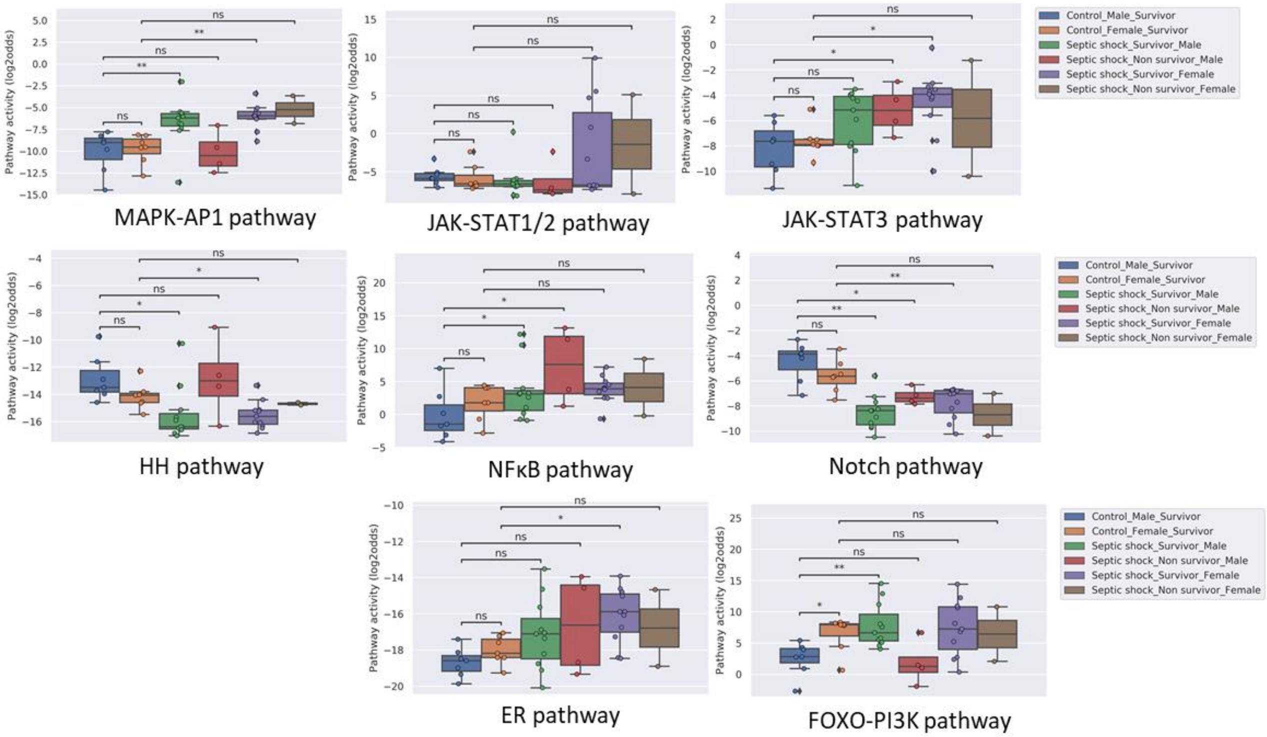

Figure S6: PAS for pathways NFκB, Notch, ER, FOXO-PI3K, HH, MAPK-AP1, JAK-STAT3 and JAK-STAT1/2 of dataset GSE9692 (5). *Two sided Mann–Whitney–Wilcoxon statistical tests were performed; p-values are indicated in the figures as *p <0.05, **p < 0.01, ***p < 0.001, ****p < 0.0001 or ns.*

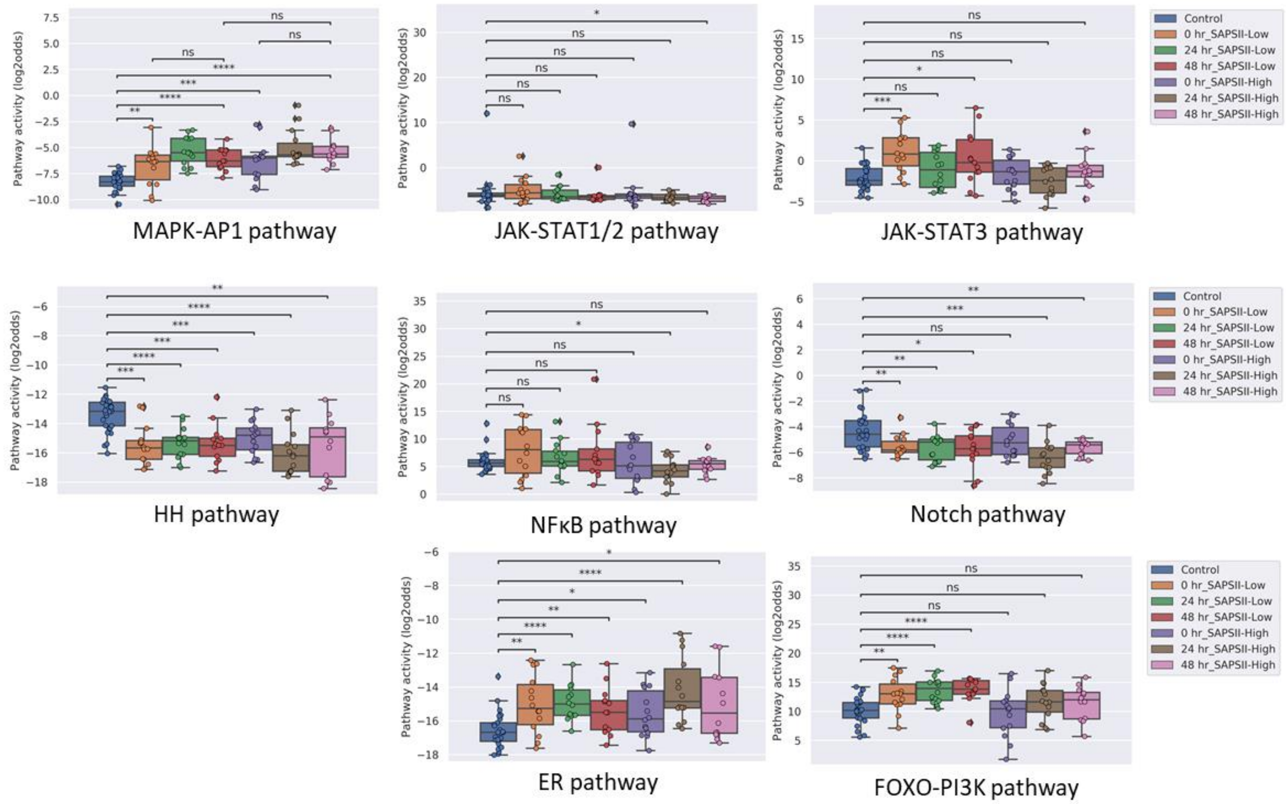

Figure S7: PAS for pathways NFκB, Notch, ER, FOXO-PI3K, HH, MAPK-AP1, JAK-STAT3 and JAK-STAT1/2 of dataset GSE57065 (6)*. Two sided Mann–Whitney–Wilcoxon statistical tests were performed; p-values are indicated in the figures as *p<0.05, **p < 0.01, ***p < 0.001, ****p < 0.0001 or ns.*

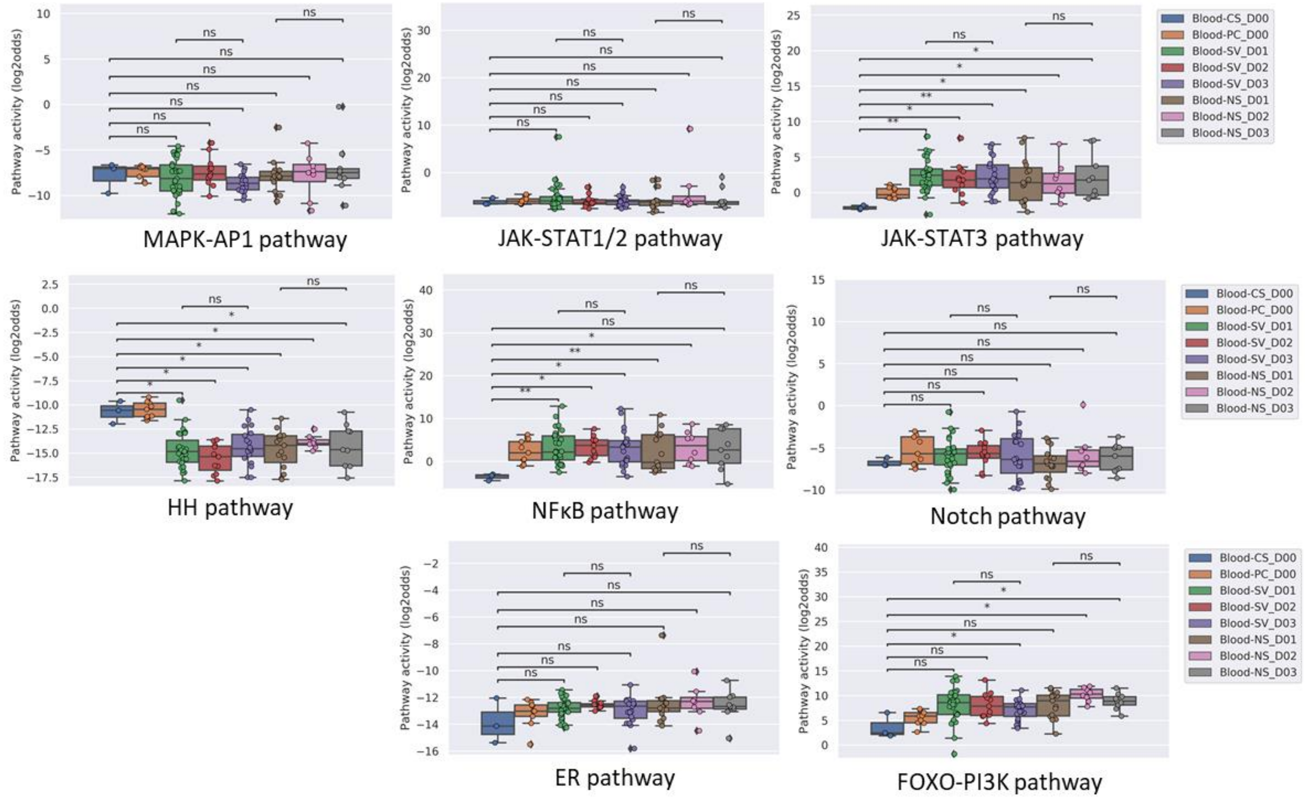

Figure S8: PAS for pathways NFκB, Notch, , ER, FOXO-PI3K, HH, MAPK-AP1, JAK-STAT3 and JAK-STAT1/2 of dataset GSE95233 (7). *Two sided Mann–Whitney–Wilcoxon statistical tests were performed; p-values are indicated in the figures as *p <0.05, **p < 0.01, ***p < 0.001, ****p < 0.0001 or ns.*

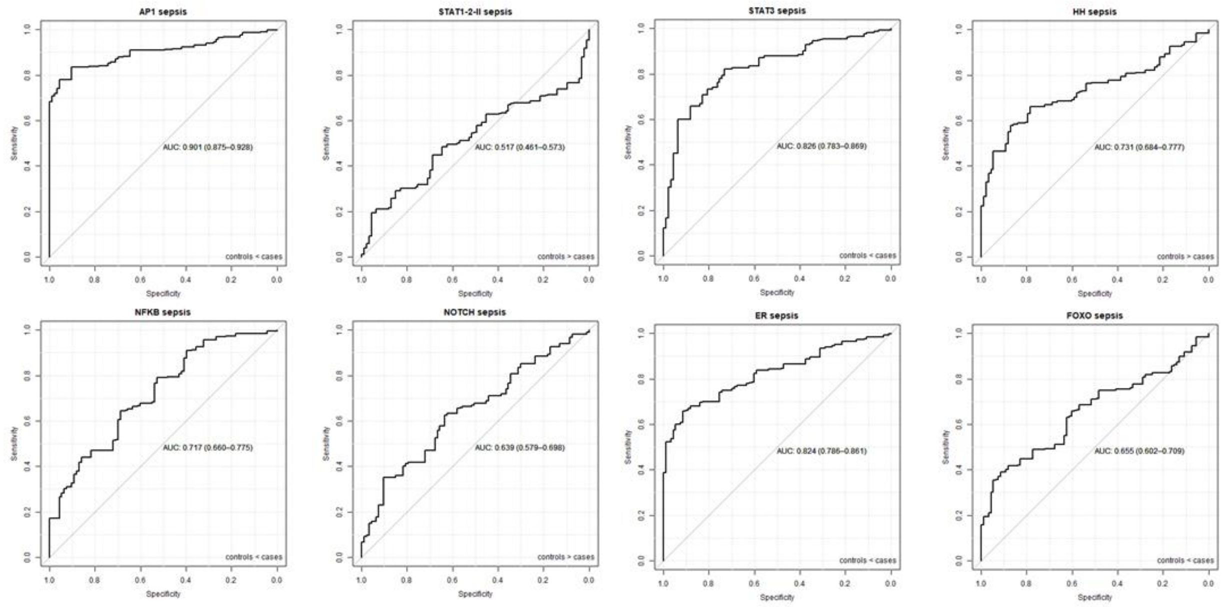

Figure S9: ROC curves for diagnosis of sepsis for pathways NFκB, Notch, TGFβ, ER, FOXO-PI3K, HH, MAPK-AP1, JAK-STAT3 and JAK-STAT1/2. Controls and sepsis patients (sepsis and sepsis shock) from dataset GSE26440 (1), GSE26378 (1), GSE4607 (2), GSE13904 (3), GSE8121(4) and GSE9692 (5) *are included.*

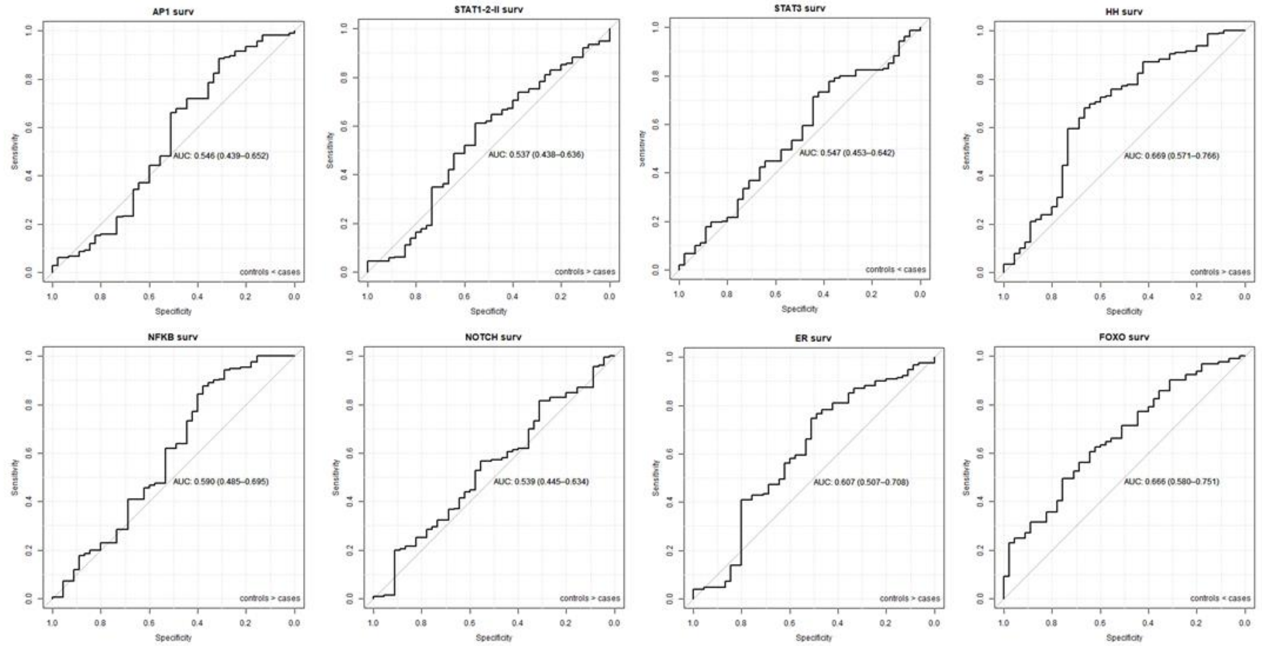

Figure S10: ROC curves for survival of sepsis for pathways NκKB, Notch, TGFβ, ER, FOXO-PI3K, HH, MAPK-AP1, JAK-STAT3 and JAK-STAT1/2 sepsis survival and non survival patients from dataset GSE26440 (1)*, GSE26378* (1)*, GSE4607* (2) *and GSE9692* (5) *are included.*

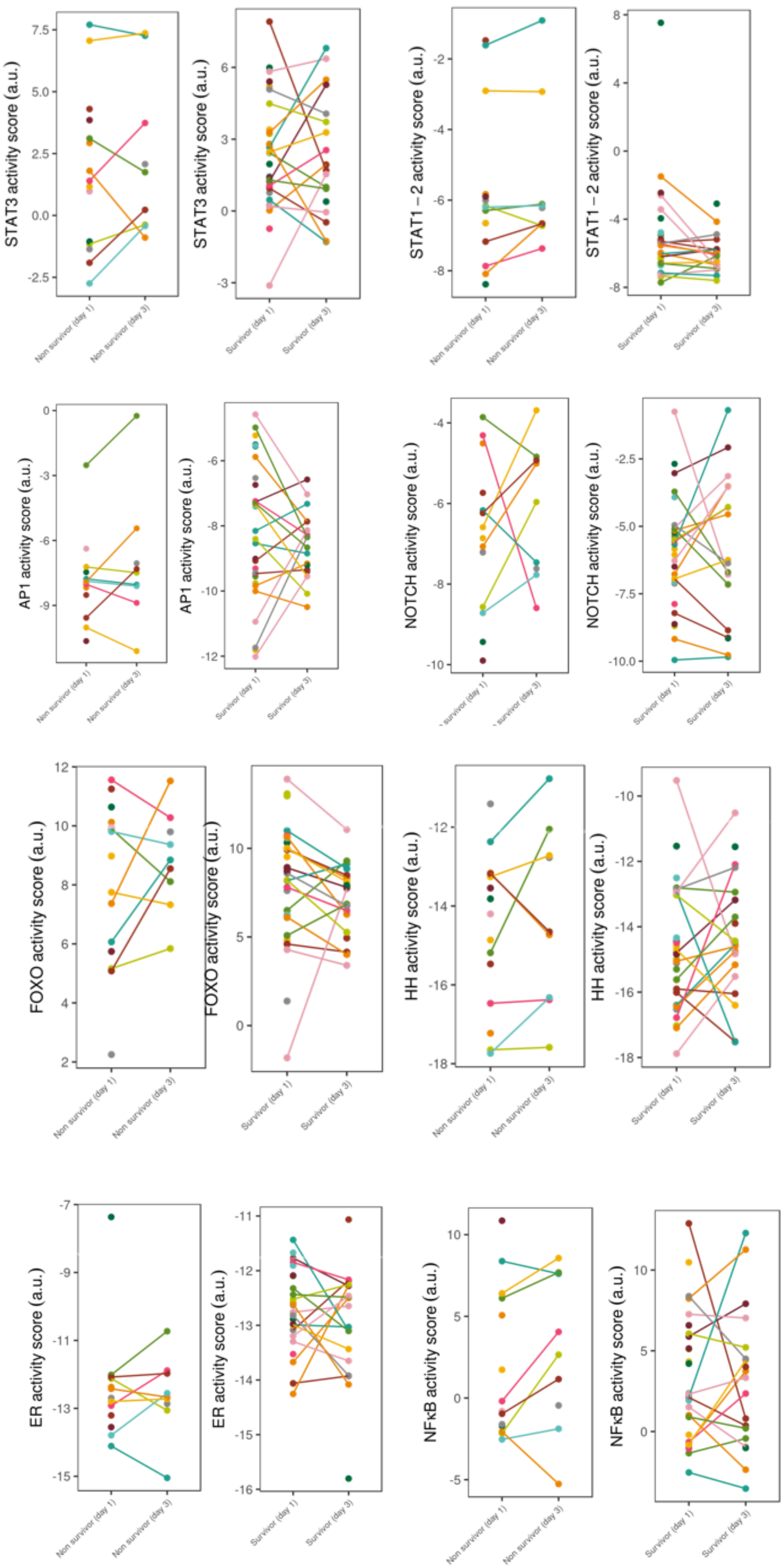

STAT3 activity score (a.u.)
STAT1 – 2 activity score (a.u.)
AP1 activity score (a.u.)
NOTCH activity score (a.u.)
FOXO activity score (a.u.)
HH activity score (a.u.)
ER activity score (a.u.)
NFκB activity score (a.u.)
Non survivor (day 1)
Non survivor (day 3)
Survivor (day 1)
Survivor (day 3)

Figure S11: PAS of day 1 versus day 3 of sepsis survival and non-survival patients for pathways NFκB, Notch, TGFβ, ER, FOXO-PI3K, HH, MAPK-AP1, JAK-STAT3 and JAK-STAT1/2. Dataset GSE95233 (7).

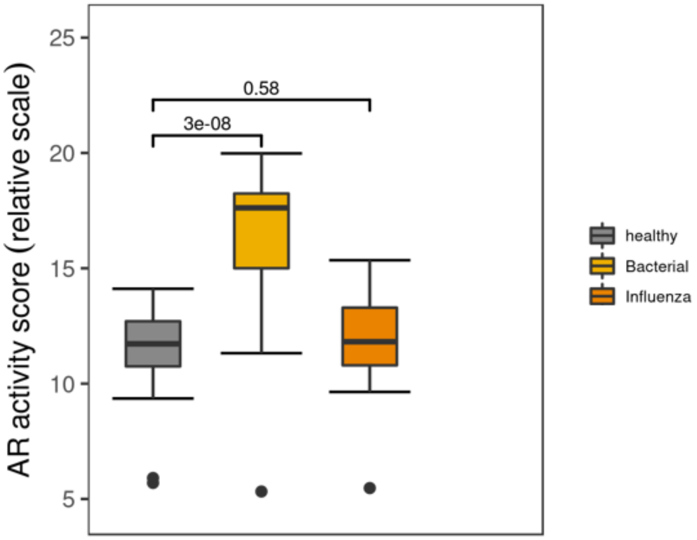

Figure S12: AR pathway activity analysis of dataset GSE161731 (8) of whole blood samples from patients presenting to the emergency department with acute respiratory infection caused by influenza (virus), or bacterial pneumonia and matched healthy controls. RNAseq data were analyzed with our signal transduction pathway assays converted to RNAseq as input data. *Two sided Mann–Whitney–Wilcoxon statistical tests is performed; p-values are indicated in the figures.*

	Healthy controls Children (log2 odds scores)			
**Pathway**	**Mean**	**SD**	**Mean + 1 SD**	**Mean + 2 SD**
MAPK-AP1	-9.67	1.73	-7.94	-6.20
AR	-15.37	1.52	-13.85	-12.33
ER	-18.22	1.10	-17.12	-16.02
HH*	-13.86	1.45	-15.31	-16.76
NFκB	1.49	3.01	4.50	7.51
Notch	-5.61	1.78	-3.84	-2.06
FOXO-PI3K	5.12	3.33	8.45	11.77
JAK-STAT1/2	-5.54	2.34	-3.20	-0.86
JAK-STAT3	-7.86	2.00	-5.86	-3.85
TGFβ	-17.05	1.63	-15.42	-13.79

	Healthy controls Adults (log2 odds scores)			
**Pathway**	**Mean**	**SD**	**Mean + 1 SD**	**Mean + 2 SD**
MAPK-AP1	-8.04	0.94	-7.10	-6.16
AR	-12.44	1.44	-11.01	-9.57
ER	-15.53	1.88	-13.65	-11.77
HH*	-12.51	1.73	-10.78	-9.04
NFκB	4.29	3.50	7.79	11.29
Notch	-4.74	1.62	-3.12	-1.50
FOXO-PI3K	8.49	3.22	11.70	14.92
JAK-STAT1/2	-5.59	3.12	-2.47	0.65
JAK-STAT3	-1.61	1.54	-0.07	1.47
TGFβ	-13.01	1.83	-11.18	-9.35

	Healthy controls Children + Adults (log2 odds scores)			
**Pathway**	**Mean**	**SD**	**Mean + 1 SD**	**Mean + 2 SD**
MAPK-AP1	-9.21	1.71	-7.49	-5.78
AR	-14.54	2.00	-12.54	-10.54
ER	-17.46	1.83	-15.63	-13.80
HH*	-13.48	1.65	-15.13	-16.77
NFκB	2.29	3.39	5.68	9.06
Notch	-5.36	1.77	-3.59	-1.82
FOXO-PI3K	6.08	3.62	9.70	13.32
JAK-STAT1/2	-5.56	2.57	-2.98	-0.41
JAK-STAT3	-6.08	3.40	-2.69	0.71
TGFβ	-15.90	2.49	-13.41	-10.92

Supplementary Table 2: Mean plus 1 and 2 SD of pathway activity scores in healthy children (n=93; GSE26440, GSE26378, GSE4607, GSE9692, GSE8121 and GSE13904) and adults (n=37; GSE57065 and GSE95233). *for HH mean minus SD.

T-test one sided	Survivors day 1 vs day 3			Non survivors day 1 vs day 3		
	paired			paired		
	p-value	significance	numbers day 1 / day 3	p-value	significance	numbers day 1 / day 3
MAPK-AP1	0.37	-	17/17	0.82	-	8/8
AR	0.19	-	17/17	0.06	-	8/8
ER	0.36	-	17/17	0.73	-	8/8
FOXO-PI3K	0.23	-	17/17	0.85	-	8/8
HH	0.67	-	17/17	0.79	-	8/8
NFκB	0.57	-	17/17	0.92	-	8/8
Notch	0.57	-	17/17	0.67	-	8/8
JAK-STAT1/2	0.06	-	17/17	0.93	-	8/8
JAK-STAT3	0.58	-	17/17	0.73	-	8/8
TGFβ	0.40	-	17/17	0.61	-	8/8

T-test one sided	Survivors day 1 vs day 3			Non survivors day 1 vs day 3		
	unpaired			unpaired		
	p-value	significance	numbers day 1 / day 3	p-value	significance	numbers day 1 / day 3
MAPK-AP1	0.12	-	31/19	0.74	-	15/9
AR	0.03	*	31/19	0.31	-	15/9
ER	0.24	-	31/19	0.41	-	15/9
FOXO-PI3K	0.11	-	31/19	0.79	-	15/9
HH	0.79	-	31/19	0.67	-	15/9
NFκB	0.43	-	31/19	0.60	-	15/9
Notch	0.50	-	31/19	0.79	-	15/9
JAK-STAT1/2	0.09	-	31/19	0.62	-	15/9
JAK-STAT3	0.44	-	31/19	0.66	-	15/9
TGFβ	0.05	-	31/19	0.49	-	15/9

Supplementary Table 3: Paired and unpaired one sided t-test for survivors and non survivors day 1 vs day 3 from dataset GSE95233. **p <0.05, **p < 0.01, - not significant.*

	Mean							
	Children						Adults	
Pathway	GSE4607	GSE26440	GSE8121	GSE9692	GSE13904	GSE26378	GSE57065	GSE95233
MAPK-AP1	-9.8	-10.1	-9.6	-9.3	-9.5	-9.1	-8.3	-7.5
AR	-15.3	-15.2	-15.3	-15.3	-15.7	-15.6	-13.1	-11.0
ER	-18.4	-18.6	-18.4	-18.0	-18.7	-17.0	-16.6	-13.3
HH	-13.6	-14.2	-13.4	-13.3	-13.6	-14.4	-13.4	-10.6
NFκB	0.8	1.0	1.0	2.0	1.0	3.4	6.0	0.8
Notch	-5.1	-5.9	-5.1	-5.0	-6.2	-5.9	-4.3	-5.7
FOXO-PI3K	4.9	4.9	4.6	5.1	5.1	6.1	10.2	5.0
JAK-STAT1/2	-5.7	-5.6	-5.5	-5.6	-5.8	-5.0	-5.4	-5.9
JAK-STAT3	-7.7	-7.5	-8.1	-8.2	-8.1	-8.3	-2.1	-0.6
TGFβ	-17.0	-17.4	-17.1	-16.6	-16.8	-16.8	-13.2	-12.6

	SD							
	Children						Adults	
Pathway	GSE4607	GSE26440	GSE8121	GSE9692	GSE13904	GSE26378	GSE57065	GSE95233
MAPK-AP1	1.9	2.0	1.7	1.5	1.2	1.5	0.8	1.0
AR	1.4	1.8	1.2	1.3	1.3	1.7	1.2	0.6
ER	0.9	1.1	0.9	0.9	1.0	0.8	1.1	1.2
HH	1.5	1.4	1.6	1.8	1.6	1.0	1.2	0.9
NFκB	3.2	3.1	3.3	3.3	2.5	1.8	1.9	3.5
Notch	2.0	1.6	1.5	1.6	2.0	1.5	1.4	1.6
FOXO-PI3K	1.1	2.6	1.2	1.3	1.3	2.4	2.2	1.9
JAK-STAT1/2	1.9	2.3	1.8	2.0	2.1	1.5	3.8	0.7
JAK-STAT3	1.6	1.6	1.4	1.0	1.3	2.3	1.5	1.2
TGFβ	3.0	3.1	2.3	2.2	3.1	3.1	2.1	1.2

Supplementary Table 4: Mean and standard deviation PAS of each dataset including healthy children and adults.

Sepsis diagnosed	sepsis confirmed	No sepsis	
Test positive	308	3	311
Test negative	91	90	181
	399	93	492

Sensitivity	77%
Specificity	97%
PPV	99%
NPV	50%
Fisher's Exact Test	p-value < 2.2e-16

Dying from sepsis	sepsis nonsurvival	sepsis survival	
Test positive	42	157	199
Test negative	3	53	56
	45	210	255

Sensitivity	93%
Specificity	25%
PPV	21%
NPV	95%
Fisher's Exact Test	p-value = 0.005023

Surviving sepsis	sepsis survival	sepsis non survival	
Test positive (below threshold)	53	3	56
Test negative (above threshold)	157	42	199
	210	45	255

Sensitivity	25%
Specificity	93%
PPV	95%
NPV	21%
Fisher's Exact Test	p-value = 0.005023

Supplementary table 6: TGFβ sensitivity, specificity, PPV and NPV.

Sepsis diagnosed	sepsis confirmed	No sepsis	
Test positive	254	2	256
Test negative	145	91	236
	399	93	492

Sensitivity	64%
Specificity	98%
PPV	99%
NPV	39%
Fisher's Exact Test	p-value < 2.2e-16

Dying from sepsis	sepsis nonsurvival	sepsis survival	
Test positive	31	142	173
Test negative	14	68	82
	45	210	255

Sensitivity	69%
Specificity	32%
PPV	18%
NPV	83%

Fisher's Exact Test p-value = 1

Surviving sepsis	sepsis survival	sepsis non survival	
Test positive (below threshold)	68	14	82
Test negative (above threshold)	142	31	173
	210	45	255

Sensitivity	32%
Specificity	69%
PPV	83%
NPV	18%
Fisher's Exact Test	p-value = 1

Supplementary table 5: AR pathway assay for sepsis diagnosis and prognosis: sensitivity, specificity, PPV and NPV.

Supplementary information (II)

Comparison of clinical study data analysis results described in previous publications coupled to the Affymetrix datasets with results obtained with STP analysis.

For each publication in which an Affymetrix dataset had been analyzed that was also analyzed for signaling pathway activity in the current study, we have listed the used bioinformatics tools for data analysis, together with obtained results with respect to functional gene annotations and identified "pathways" as defined by the used tools (Ingenuity, PANTHER, D.A.V.I.D., ToppGene), and finally the data-analysis-related conclusions taken from the associated publication abstract.

Comparing the "pathway" results with the results of the STP analysis described in the current paper illustrates the differences between the two approaches (as described before,(9), (10)). First, there is hardly or no overlap between the "pathways" identified by the use of Ingenuity/PANTHER/DAVID/ToppGene, and the signal transduction pathways that were shown in the current analysis to be abnormally active in patients with sepsis/septic shock, and related to survival. The PR, AR, ER, and TGFβ pathways were not mentioned in any of the previous analyses results. MAPK and NFkB pathways came up a few times as linked to sepsis, though their activation state remained unknown. IL6 and IL10 pathway signaling came up, which can be linked to the JAK-STAT3 pathway signaling pathway, however, no information on the activation state of the pathway was provided by the analysis. Mostly differentially expressed genes were be linked to all kinds of physiological mechanisms, by means of functional annotation, or to immune processes like T cell or B cell activation, dendritic cell maturation, etc (see below).

Though conceivable that such cellular processes may be more prominent in patients with sepsis, and lead to new hypothesis regarding the pathophysiology, their description is vague. Identification does not directly lead to new therapeutic approaches, since these cellular processes do not present targets for existing drugs/treatments, and therefore are not clinically actionable. Also the effect of the drug on such processes cannot be measured, especially not in a quantitative manner. Similarly, they may provide leads to develop diagnostic/prognostic tests, however there remains a long track for validation of any of the gene classifiers. This includes development of an mRNA-based assay which is applicable to a single sample (as is the case for the STP assays).

Sofar, to the best of our knowledge, none of such classifiers made it to clinical implementation.

As an example, while a carefully validated (on an independent dataset) relation between low Zn levels and sepsis was found (5), the clinical relevance of zinc levels for prognosis of sepsis patients has subsequently not been confirmed in clinical studies and current guidelines for pediatric sepsis treatments advise against zinc administration in view of lack of clinical evidence (11). Thus, while bioinformatics analysis revealed a robust *association* between decreased Zn levels and sepsis, a causal relation was probably lacking. This resulted in a negative clinical study and negative advice with respect to Zn supplementation.

In summary, bioinformatics tools such as Ingenuity, define a “pathway” as “any cellular (biochemical) process”, and have been developed for gene expression-based *biomarker discovery purposes* by comparing groups of sample data, and as such, for hypothesis

generation. Development of diagnostic assays or identification of a new drug target can start from there.

Summary identified signaling pathways (expression of signaling pathway components, without indication about pathway activity status):

1. *NFκB / TLR (associated with NFκB pathway):* (1),(4),(5),(2)
2. *P38-MAPK /PDGF (associated with MAPK-AP1 pathway):* (4),(5),(6),(2)
3. *IL6/IL10 (associated with JAK-STAT3 pathway):* (4),(5),(6),(2)
4. *Integrin signaling/Insulin signaling/IGF1 signaling (associated with PI3K pathway):* (4)
5. *Wnt (associated with the Wnt pathway):* (4)

<u>Description of bioinformatics tools used and obtained results per clinical study</u>

J. L. Wynn *et al.*, "The influence of developmental age on the early transcriptomic response of children with septic shock," *Mol Med*, vol. 17, no. 11–12, pp. 1146–1156, 2011, doi: 10.2119/molmed.2011.00169. Dataset GSE26440 (GSE26378)

Bioinformatics tools used to identify cellular pathways (not specifically signal transduction pathways)

- Ingenuity

Identified "pathways" in pediatric sepsis patients:

1. B-cell receptor signaling
2. TREM1 signaling
3. Pattern recognition receptor signaling
4. NF-κB signaling
5. Dendritic cell maturation
6. Communication between innate and adaptive immunity

Conclusions taken from publication-abstract:

- Sepsis: reduced expression of genes representing key pathways of innate and adaptive immunity
- Sepsis: predominantly downregulated transcriptome
- Sepsis: Neonates and school-age subjects had the most uniquely regulated genes relative to controls.

General: "Age-specific studies of the host response are necessary to identify developmentally relevant translational opportunities that may lead to improved sepsis outcomes."

H. R. Wong *et al.*, "Genome-level expression profiles in pediatric septic shock indicate a role for altered zinc homeostasis in poor outcome," *Physiol Genomics*, vol. 30, no. 2, pp. 146–155, Jul. 2007, doi: 10.1152/physiolgenomics.00024.2007. Dataset GSE4607

Bioinformatics tools used to identify cellular pathways (not specifically signal transduction

- D.A.V.I.D. (Database for Annotation, Visualization and Integrated Discovery

- Ingenuity Pathway Analysis, limited to “canonical pathways”

Results data analysis

D.A.V.I.D.: top functional gene annotations, upregulated genes:

1. direct protein sequencing
2. response to other organism
3. response to pest/pathogen/parasite
4. glycoprotein
5. response to stress
6. membrane
7. response to biotic stimulus
8. response to wounding
9. signal
10. inflammatory response
11. response to external stimulus
12. defense response
13. immune response
14. protein binding
15. lipoprotein
16. phosphorylation
17. intracellular signaling cascade
18. plasma membrane

19. signal transduction

20. protein kinase cascade

D.A.V.I.D.: Top 20 functional annotations, downregulated genes in patients with septic shock

1. nuclear protein
2. zinc
3. zinc-finger
4. transcription
5. nucleus
6. transcription regulation
7. dna-binding
8. metal-binding
9. t-cell
10. nucleic acid binding
11. zinc ion binding
12. membrane-bound organelle
13. regulation of intracellular physiological process
14. regulation of cellular process
15. defense response
16. nucleobase, nucleoside, nucleotide, and nucleic acid metabolism
17. regulation of physiological process
18. immune response
19. antigen processing/presentation

20. regulation of biological process

Ingenuity Pathways analysis (canonical): upregulated genes

1. Interleukin-6 signaling
2. Interleukin-10 signaling
3. Toll-like receptor signaling
4. B-cell receptor signaling
5. Integrin signaling
6. Complement and coagulation cascades
7. Granulocyte/macrophage-colony stimulation factor signaling
8. p38 MAP kinase signaling
9. Leukocyte extravasation signaling
10. NF-κB signaling

Ingenuity Pathways Analysis (canonical), downregulated genes:

1. T-cell receptor signaling
2. Antigen presentation pathway
3. Natural killer cell signaling
4. Cell Cycle: G1/S checkpoint regulation
5. N-glycan biosynthesis

Conclusions taken from publication-abstract:

- genome-level alterations of zinc homeostasis

H. R. Wong *et al.*, "Genomic expression profiling across the pediatric systemic inflammatory response syndrome, sepsis, and septic shock spectrum," *Crit Care Med*, vol. 37, no. 5, pp. 1558–1566, May 2009, doi: 10.1097/CCM.0b013e31819fcc08. Dataset GSE13904.

Bioinformatics tools used to identify cellular pathways (references in publication)

- D.A.V.I.D. (Database for Annotation, Visualization and Integrated Discovery
- PANTHER Classification System
- ToppGene

Results data analysis

Functional annotation at day 1 sepsis:

PANTHER

- Pathway: T-cell activation (5.1E–15)
- Biological process: MHC II-mediated immunity (1.8E–17)
- Molecular function: major histocompatibility complex antigen (5.5E–14)

ToppGene

- Molecular function: MHC class II receptor activity (<1.0E–6)
- Biological process: antigen processing and presentation (<1.0E–6)
- Mouse phenotype: abnormal antigen processing via MHC class II (<1.0E–6)
- Pathway: antigen processing and presentation (<1.0E–6)

D.A.V.I.D.

- MHC class II receptor activity (1.6E–9)

Functional annotation at day 3 sepsis:

PANTHER

- Pathway: T-cell activation
- Biological process: T-cell–mediated immunity
- Molecular function: major histocompatibility complex antigen

ToppGene

- Molecular function: MHC class II receptor activity
- Biological process: antigen processing via MHC class II
- Mouse phenotype: abnormal immune system physiology
- Pathway: antigen processing and presentation

D.A.V.I.D.

- MHC class II receptor activity

Conclusions from publication-abstract:

- septic shock: repression of genes corresponding to adaptive immunity and zinc-related biology.
- Causality remains to be elucidated.

T. P. Shanley *et al.*, "Genome-level longitudinal expression of signaling pathways and gene networks in pediatric septic shock," *Mol Med*, vol. 13, no. 9–10, pp. 495–508, Oct. 2007, doi: 10.2119/2007-00065. Dataset GSE8121

Bioinformatics tools for pathway analysis

- Ingenuity
- D.A.V.I.D.
- PANTHER

Results data analysis

Ingenuity Pathways Analysis, upregulated genes:

Day 1 and day 3 septic shock:

- B Cell Receptor Signaling
- GMCSF signaling
- IL10 signaling
- NFkB signaling
- p38 MAPK signaling
- Complement and coagulation
- TLR signaling
- IL6 signaling

Only day 3 septick shock:

- Integrin signaling
- IGF1 signaling

- Insulin Receptor Signaling
- PPAR signaling

Ingenuity Pathways Analysis, downregulated genes

- Antigen presentation
- NK cell signaling
- T cell receptor signaling

PANTHER Gene Networks, upregulated genes:

Only day 1 sepsis:

- Oxidative stress response
- Inflammation mediated by chemokine and cytokine signaling
- PDGF signaling

Only day 3 sepsis

- Inflammation mediated by chemokine and cytokine signaling
- Toll receptor signaling pathway
- Plasminogen activating cascade
- PDGF signaling pathway
- Insulin/IGF pathway-protein kinase B signaling pathway

PANTHER Gene Networks, downregulated genes:

Only day 1 sepsis:

- T cell signaling

- Wnt signaling pathway

Only Day 3 sepsis:

- T cell activation
- B cell activation

Conclusions from publication- abstract:

- Differential regulation of genes involved in multiple signaling pathways and gene networks primarily related to immunity and inflammation.
- distinct gene networks involving T cell- and MHC antigen-related biology were downregulated on both day one and day three.
- downregulation of genes corresponding to functional annotations related to zinc homeostasis.

General: The data further advance our genome-level understanding of pediatric septic shock and support novel hypotheses.

N. Cvijanovich *et al.*, "Validating the genomic signature of pediatric septic shock," *Physiol Genomics*, vol. 34, no. 1, pp. 127–134, Jun. 2008, doi: 10.1152/physiolgenomics.00025.2008. Dataset GSE9692

Bioinformatics tools used for data analysis

- Ingenuity Pathways Analysis
- PANTHER

Results data analysis

Ingenuity Pathways Analysis, upregulated genes

1. Toll-like receptor signaling
2. Interleukin-10 signaling
3. NF-κB signaling
4. Acute phase response signaling
5. p38 MAP kinase signaling
6. Complement system
7. Hepatic cholestasis
8. LXR/RXRα activation
9. Interleukin-6 signaling
10. PPARα/RXRα activation

Ingenuity Pathways Analysis, Down-regulated genes

- Natural killer cell signaling
- T-cell receptor signaling
- Antigen presentation pathway
- Interleukin-4 signaling

Gene selection from the original reported gene list (2) led to a 50 gene predictor gene classifier.

Using Ingenuity, this classifier was found to be related to:

- NF-κB signaling
- Leukocyte extravasation signaling
- Liver X receptor/Retinoid X receptor signaling

- T cell receptor signaling
- p38 MAP kinase signaling

Conclusions from publication-abstract:

- validation cohort: repression of genes related to zinc homeostasis and lymphocyte function

M.-A. Cazalis *et al.*, "Early and dynamic changes in gene expression in septic shock patients: a genome-wide approach," *Intensive Care Medicine Experimental*, vol. 2, no. 1, p. 20, Aug. 2014, doi: 10.1186/s40635-014-0020-3. Dataset GSE57065

Bioinformatics tools used for data analysis

- Ingenuity Pathways Analysis

Results data analysis

Downregulated pathways

1. Calcium-induced T lymphocyte Apoptosis
2. iCOS-iCOSL signaling in T helper cells
3. Cytotoxic T lymphocyte mediated apoptosis of target cells
4. PKCO Signalingin T lymphocytes
5. Role of NFAT in regulation of the immune response
6. Nur77 signaling in T lymphocytes
7. CD28 signaling in T helper cells
8. OX signaling pathway

9. Allograft rejection signaling
10. B cell development

Upregulated pathways:

1. Hepatic fibrosis/Hepatic stellate activation
2. P38MAPK signaling
3. IL10 signaling
4. LXR/RXR activation
5. Complement system
6. O Glycan Biosynthesis
7. NOS signaling
8. Glutathione metabolism
9. Hypoxia signaling in the cardiovascular system
10. IL-6 signaling

Conclusions from abstract

- both pro- and anti-inflammatory processes are induced within the very first hours after septic shock.
- more severe patients did not exhibit the strongest modulation.
- "This reinforces the idea that an immediate and tailored aggressive care of patients, aimed at restoring an appropriately regulated immune response, may have a beneficial impact on the outcome."

F. Venet *et al.*, "Modulation of LILRB2 protein and mRNA expressions in septic shock patients and after ex vivo lipopolysaccharide stimulation," *Hum Immunol*, vol. 78, no. 5–6, pp. 441–450, Jun. 2017, doi: 10.1016/j.humimm.2017.03.010. Dataset GSE95233

Bioinformatics tools used for data analysis: none. Differential expression analysis identified LILRB2 (leukocyte immunoglobulin-like receptors subfamily B, member 2) as higher expressed in sepsis.

Conclusion from publication-abstract:

- LILRB2 protein and mRNA expressions are deregulated on monocytes after septic shock

Dataset GSE11755 (2008). No associated publication.

Conclusion from GEO information (not published since 2008):

- Definition of an expression profile for meningococcal sepsis.

References

1. Wynn JL, Cvijanovich NZ, Allen GL, Thomas NJ, Freishtat RJ, Anas N, et al. The influence of developmental age on the early transcriptomic response of children with septic shock. Mol Med. 2011;17(11–12):1146–56.

2. Wong HR, Shanley TP, Sakthivel B, Cvijanovich N, Lin R, Allen GL, et al. Genome-level expression profiles in pediatric septic shock indicate a role for altered zinc homeostasis in poor outcome. Physiol Genomics. 2007 Jul 18;30(2):146–55.

3. Wong HR, Cvijanovich N, Allen GL, Lin R, Anas N, Meyer K, et al. Genomic expression profiling across the pediatric systemic inflammatory response syndrome, sepsis, and septic shock spectrum. Crit Care Med. 2009 May;37(5):1558–66.

4. Shanley TP, Cvijanovich N, Lin R, Allen GL, Thomas NJ, Doctor A, et al. Genome-Level Longitudinal Expression of Signaling Pathways and Gene Networks in Pediatric Septic Shock. Mol Med. 2007;13(9–10):495–508.

5. Cvijanovich N, Shanley TP, Lin R, Allen GL, Thomas NJ, Checchia P, et al. Validating the genomic signature of pediatric septic shock. Physiol Genomics. 2008 Jun 12;34(1):127–34.

6. Cazalis M-A, Lepape A, Venet F, Frager F, Mougin B, Vallin H, et al. Early and dynamic changes in gene expression in septic shock patients: a genome-wide approach. Intensive Care Med Exp. 2014 Dec;2(1):20.

7. Venet F, Schilling J, Cazalis M-A, Demaret J, Poujol F, Girardot T, et al. Modulation of LILRB2 protein and mRNA expressions in septic shock patients and after ex vivo lipopolysaccharide stimulation. Hum Immunol. 2017 Jun;78(5–6):441–50.

8. McClain MT, Constantine FJ, Henao R, Liu Y, Tsalik EL, Burke TW, et al. Dysregulated transcriptional responses to SARS-CoV-2 in the periphery. Nature Communications. 2021 Feb 17;12(1):1079.

9. Verhaegh W, van Ooijen H, Inda MA, Hatzis P, Versteeg R, Smid M, et al. Selection of personalized patient therapy through the use of knowledge-based computational models that identify tumor-driving signal transduction pathways. Cancer Res. 2014 Jun 1;74(11):2936–45.

10. van de Stolpe A, Holtzer L, Ooijen H van, Inda MA de, Verhaegh W. Enabling precision medicine by unravelling disease pathophysiology: quantifying signal transduction pathway activity across cell and tissue types. Scientific Reports. 2019 Feb 7;9(1):1603.

11. Weiss SL, Peters MJ, Alhazzani W, Agus MSD, Flori HR, Inwald DP, et al. Surviving sepsis campaign international guidelines for the management of septic shock and sepsis-associated organ dysfunction in children. Intensive Care Med. 2020 Feb 1;46(1):10–67.
